# The Effect of Pranayamic Breathing as a Positive Psychology Exercise on Foreign Language Learning Anxiety and Test Anxiety Among Language Learners at Tertiary Level

**DOI:** 10.3389/fpsyg.2021.742060

**Published:** 2021-09-30

**Authors:** Murselin Tasan, Enisa Mede, Karim Sadeghi

**Affiliations:** ^1^Foreign Languages School, Istanbul Medipol University, Istanbul, Turkey; ^2^ELT Department, Bahçeşehir University, Istanbul, Turkey

**Keywords:** pranayamic breathing, mindfulness, test anxiety, foreign language anxiety, mindfulness-based stress reduction, positive psychology

## Abstract

This study investigated the impact of pranayamic breathing (PB) as a positive psychology exercise on mitigating foreign language anxiety (FLLA) and test anxiety (TA) of undergraduate English students studying at a Turkish university. Additionally, the study examined the effects of PB on academic achievement in listening and reading comprehension skills of the participants as well as exploring learners’ and their instructor’s perceptions of using PB techniques in their classrooms. The sample consisted of 140 sophomore English language learners. Two intact classes, each comprising 70 participants, were selected as the experimental and the control group using convenience sampling. Two basic PB techniques, Nadi Shodhana Pranayama and Bhramari Pranayama, were implemented to the experimental group on a weekly basis for a total of 7 weeks. In this mixed-method study, the quantitative data were gathered using English Language Learning Anxiety Scale, Foreign Language Test Anxiety Scale, and listening and reading achievement pre- and post-tests, while the qualitative data were collected using semi-structured interviews, and the teacher’s reflective journal. The findings revealed that the implementation of positive psychology technique of pranayama breathing mitigated the FLLA and TA levels significantly and also improved listening and reading comprehension skills of the participants to a considerable extent. The findings also demonstrated that both the students and their instructor perceived PB implementation as a useful and a practical medium in alleviating the anxious feelings, promoting the general class atmosphere and regulating daily habits despite the implementational challenges shared by the instructor.

## Introduction

The world keeps changing at a rate that has become impossible to catch up with; and with each passing day, the number of the people struggling with the psychophysiological burdens of this fast-paced environment is on the rise. As an inevitable consequence of these rapid alterations in everyday life, the words stress, and anxiety are now perceived as eerie and catastrophic incidents. In fact, a majority the disorders credited to stress and anxiety are comorbid and many victims do not seek professional help or even get the right treatment (Tiller, 2012). As reported by World Health Organization (WHO), the total number of people burdened with depression is more than 300 million as of 2015 and the approximate number of anxiety disorder victims is nearly the same as the depression cases (World Health Organization, 2017). Specifically, anxiety is portrayed as the embodiment of tense conditions that are rooted in the autonomic brain system activities (Scovel, 1978) and the exploitation of rational thinking. It is depicted as a mental reaction to obscure fear and distress (Middeldorp et al., 2005). Regardless of its various delineations, anxiety continues to victimize a growing number of worldwide population irrespective of age, gender, and socio-economic conditions (Craske and Stein, 2016).

By the same token, learners in the process of acquiring a foreign language are no exception to anxiety’s impact. Whether it is low, mild, or severe, foreign language learning anxiety (FLLA) is notoriously famous for affecting the language learning process negatively and is one of the densely examined topics in educational research (Horwitz, 2001). Hashemi (2011) claims that English language learners frequently report feeling stressed out or anxious when performing the language and they might even refrain from the learning process due to anxiety provoking feelings. Furthermore, Dörnyei (2005) emphasizes that the presence of anxiety may result in learners’ incapability to perform the language even though they are cognitively capable of acquiring it. In line with FLLA, test anxiety (TA) also appears to be one of the salient factors influencing the quality of the learning process. Although some learners might feel relaxed while performing the foreign language, they might think they have done poorly in examination settings and thus become the victims of anxiety (Young, 1990). If not interfered, this kind of debilitating anxiety can result in demotivation of learners, who may accordingly be reluctant to perform language tasks and show avoidance attitudes (Scovel, 1978).

At this juncture, the role of pranayamic breathing (PB) as a positive psychology exercise manifests itself as a solvent assistance to mitigate the levels of this debilitative worrying. With its long history dating back to before common era, pranayama might offer forbearing mindfulness-based anxiety reducing solutions to its practitioners in order to enhance the challenging nature of the learning conditions (Butzer et al., 2016b). Pranayama, which is a vital component in yogic discipline, is translated from Sanskrit as the regulation of breath and the control of life energy (James, 2009). Both yoga and positive psychology center around the transformation of individuals for a more meaningful and a fulfilling existence that entails higher levels of wellbeing and spirituality (Butzer et al., 2016b). The concept of “*prana* (life energy)” and “*yama* (disciplining one’s self)” have caught much attention in the field of positive psychology so far (Ivtzan and Papantoniou, 2014). It has also been highlighted that positive psychology and pranayama share the same notions when explaining mindfulness, which are the importance of self-control, self-discipline, and self-awareness, remaining engaged in a task and staying at the moment (Ivtzan and Papantoniou, 2014). Therefore, yoga and its components are valued as the quintessential positive psychologies and viewed as providing fundamental frameworks in positive psychology (Levine, 2000).

## Literature Review

Yoga and its diverse implications have received substantial attention throughout the world with the general assumption that yoga as a positive psychology technique has the potential to nurture the overall psychological being (Singleton, 2010). Standing out as a long-standing mindfulness-based discipline in yoga discipline and defined as “a receptive attention to and awareness of present events and experience” (Brown et al., 2007, p. 212), pranayama as a positive psychology exercise enhances the psychological and physiological being and ameliorates standards of life (Kharya et al., 2014). Moreover, the effects of yogic breathing techniques are known to be lasting for longer periods of time and no single set up or materials are needed to benefit from the impacts of pranayama (Anand et al., 2018). Although PB has not been implemented in foreign and second language learning contexts so far, the clinical studies conducted indicate that it works in lowering down the stress levels of practitioners as well as improving other health conditions such as cardiovascular diseases, lung diseases, and various mental illnesses (Brown and Gerbarg, 2005; Anand et al., 2018; Kuppusamy et al., 2018). Due to PB’s reported benefits in reducing stress levels in other professions and in order to examine and extend its effectiveness in the context of education, this study is one of the first to experiment with this positive psychology technique in second language learning.

Foreign language anxiety (FLA) is generally perceived as an obstacle which might inhibit or debilitate the learning or the acquisition process of languages (Suleimenova, 2013). Horwitz et al. (1986) explicated FLA as a multifaceted process containing feelings, ideas, and attitudes of the individuals toward language learning. The four language skills, reading, writing, speaking, and listening have an effect on how individuals experience the learning process (Elkhafaifi, 2005). Individuals suffering from speaking anxiety may avoid participating in class discussions due to the reluctance to speak (Suleimenova, 2013). The presence of listening anxiety might affect learners’ overall ability to comprehend the intended messages and create responses accordingly (Halat and Özbay, 2018). Sellers (2000) reported that learners with high reading anxiety levels did not remember the content of the reading passages when compared to others who did not show the symptoms of reading anxiety.

The right amount of anxiety can help an individual to be successful, but observations and learner experiences indicate that TA might influence the foreign language learning process negatively (Aydın et al., 2006). von Wörde (2013) considers TA as concerns related to failure based on individuals’ performance. TA is an apprehension stemming from academic evolution, the fear of being unsuccessful or failing (Horwitz and Young, 1991). Testing techniques, the format of the tests, time limitations, or the evaluating environment have a significant impact on TA (Yue, 1996). Not being prepared enough for a language test might also startle worrisome feelings (Aydın et al., 2006). Regardless of the causes of TA, it is still a field that offers instructors more insights in understanding what kind of anxiety needs to be focused on in class. Pranayama is known to be helpful in stressful situations since it helps individuals to regulate their breathing patterns and gather focus (Brown and Gerbarg, 2005). Hence, the efficacy of PB on TA is a promising area that requires further attention.

Aligned with FLA and TA, language comprehension skills also come to the fore when explicating the foreign language acquisition process. Negative impact of stress could be immensely important in the development of listening and reading comprehension (Bloomfield et al., 2010). Among the factors influencing listening and reading comprehension are motivation, concentration, executive functioning skills, memory capacity, the usage strategies, the foreign language expertise, and anxiety (Yildiz and Albay, 2015). Considering that mindfulness breathing exercises work on enhancing memory, attention, and tranquilizing the stressful mind (Jensen et al., 2012), pranayama could aid learners in fostering listening and reading comprehension through promoting executive functioning skills. Mrazek et al. (2013) found that mindfulness-based activities decreased the mind wandering behavior of the learners due to higher levels of concentration during reading tasks. In addition, Vhavle et al. (2017) highlighted improvements in cognitive abilities such as possessing significantly better memory, attention, and reasoning capabilities as well as changes in listening and oral comprehension abilities after practicing yoga. Recognizing that both listening and reading comprehension consist of interactions, memory, lexical knowledge, motivation, metacognitive skills, and most importantly, stress, the implementation of pranayama could be promising for language learners (Meniado, 2016).

Two particular PB techniques, Nadi Shodhana Pranayama (NSP) and Bhramari Pranayama (BP), stand out as the most commonly practiced techniques and were employed in this study. Nadi Shodhana, which is a PB method that is practiced smoothly, does not demand any equipment or prior exercises to be applied (Joshi et al., 2011). NSP is usually illustrated as an alternative technique of breathing through the nostrils (Sengupta, 2012). Several studies involving NSP practices affirm the benefits observed in autonomic brain and cardiovascular functions, pulmonary disorders, and blood pressure (Dhanvijay and Chandan, 2018; Shah and Kothari, 2019). Shah and Kothari (2019) report in their study that NSP is effective in tranquilizing the stressful mind. In another study by Gupta et al. (2014) the authors found out ameliorations in problem solving skills of the participants. The word Bhramari, on the other hand, comes from the word Bhramar which means bumble bee or wasp in Sanskrit (Malhotra et al., 2016). In this PB technique, humming sounds that resemble wasps are made during exhalation process (Srivastava et al., 2017). When this PB is practiced, the ears are covered by the thumbs and the eyes are closed off with fingers. Another study by Malhotra et al. (2016) discovered statistically significant changes in the levels of concentration after practicing BP. Regardless of age and sex, BP can be applied by anyone without any difficulties (Kuppusamy et al., 2018).

Pranayama can be practiced without the need of a single setup, material or equipment and the immediate effects could be achieved very swiftly (Kumar and Pradhan, 2017; Anand et al., 2018). Moreover, the immediate benefits of pranayama could be witnessed even in practices that take up to 5 or 6 min (Pramanik et al., 2009). The number of studies, which were carried out to analyze the effectiveness of pranayama and yoga in decreasing stress and anxiety levels as well as improving health conditions related to lung capacity, respiratory and cardiovascular diseases, asthma, epilepsy, immune system disorders, diabetes, autonomic dysfunctions, muscular endurance, mental disorders, blood pressure, hypertension, and chronic headaches (Kharya et al., 2014; Butzer et al., 2016a; Hepburn and McMahon, 2017; Kumar and Pradhan, 2017; Kuppusamy et al., 2017) is highly abundant in the field of psychology and medicine. Although, the aforementioned studies highlighted the effects of pranayama on various physical and psychological disorders, no single research up to date has aimed at exploring the capacity of PB techniques in reducing FLLA or TA levels expect one study probing the impact of PB on TA (Nemati, 2013). Yet, Nemati’s research was carried out with learners studying disciplines that were not general English (GE) language based at post graduate levels.

The uncharted effects of PB on FLLA and TA should be analyzed thoroughly. Krashen (1982) expresses the need to create safe learning environments for the foreign language learners so that their affective filter could be lowered. Accordingly, the impact of PB on foreign language teaching contexts needs to be particularly examined in relation to the FLA since it might be rewarding to discover the hidden potentials of how PB impacts the process of foreign language learning. In addition, it is important to remember that foreign language instructors are in need to construct learning environments in which affective filter is low to aid learners in accessing comprehensible input in a safer learning atmosphere (Krashen, 1982). Nowadays, the inevitable growth of anxiety is, unfortunately, something that language learners cannot escape from. Although there have been quite a few studies carried out to find solutions to mitigate foreign language learning and performance anxiety, instructors are desperately in need of quick, long-lasting, and cost-effective solutions to help their learners. Since language acquisition takes places not only inside the classroom atmosphere but also outside, students need to transform themselves into autonomous learners and control what affects their wellbeing tremendously, an important one being the breath.

In line with what has been presented so far, the utmost goal of this study was to investigate the effectiveness of the implementation of two PB techniques, Nadi Shodhana and Bhramari, on mitigating the FLLA of undergraduate English language learners in the context of a Turkish university. The study also aimed to compare the changes observed on the TA levels of undergraduate learners over the course of PB implementation process. Additionally, the differences in the listening and reading comprehension skills of the aforementioned learners were intended to be probed by analyzing the effects of PB implementation. Ultimately, the study attempted to explore the insights that could be gained into the integration of PB in GE language classes through exploring the instructor’s and learners’ views on its effectiveness. Although it is known that FLLA and TA have been vastly probed research areas, the solutions to diminish those affective variables’ negative aspects should be more long-lasting, cost effective and practical. Herein, this study targeted at presenting a solution that has never been attempted before in English as a Foreign Language (EFL) context, the accomplishment of which still remains a big gap in the literature. The findings of this study are expected to contribute to the field of language education by introducing the steps to use PB to surpass FLLA and TA among learners, and also, practitioners may even reap the benefits of pranayama pertaining to probable remediations regulating physical and psychological disorders.

Based on these objectives, this study targets at seeking answers to the following research questions:

(1) Does the implementation of PB techniques have any impact on the FLLA of the participants?

(2) Does the implementation of PB techniques have any impact on the TA level of the participants?

(3) Is there any difference in the listening comprehension skills of the participants after being exposed to PB?

(4) Is there any difference in the reading comprehension skills of the participants after being exposed to PB?

(5) What are the perceptions of students and their breathing instructor about integrating PB in their classroom practices?

## Materials and Methods

### Participants

The participants of the study consisted of 140 undergraduate sophomore learners from the Faculty of Health Sciences at a non-profit foundation university in Istanbul, Turkey. The participants, whose age ranged from 17 to 27 (*M* = 19.78, SD = 1.62), took GE classes at pre-intermediate (A2) proficiency level. Two intact groups were selected as the experimental (*n* = 70) and the control group (*n* = 70) according to convenience sampling. The assignment of groups to experimental or control group was done randomly. Overall, 70% (*n* = 98) of the participants were female and 30% (*n* = 42) were male. Twenty participants from both control (*n* = 12) and experimental group (*n* = 8) were already familiar with basic, technical breathing exercises prior the study as they were studying Health Sciences. Fifty-six participants were exercising regularly in the control (*n* = 27) and experimental (*n* = 29) group.

### Materials

#### English Language Learning Anxiety Scale

To evaluate the impact of PB on alleviating FLA, ELLAS was applied both to the experimental and control groups before and after the implementation. The scale targeted at evaluating the FLLA including four subscales related to listening, reading, speaking, and writing anxiety each containing seven items. Overall, ELLAS included 28 items (all negatively coded) and was based upon a 5-point Likert Scale ranging from 1 (Strongly Disagree) to 5 (Strongly Agree). Some sample items were as follows: *I feel unhappy when I do not understand what my teacher and friends say in English*. *When we read texts in English, I feel overwhelmed and end up getting lost. I do not feel relaxed when I speak English. During writing in English, I feel worried since I cannot express myself clearly.* Higher scores imply higher levels of English language learning anxiety. ELLAS was piloted with a similar group from the Faculty of Health Sciences and the Cronbach’s alpha value was estimated to be 0.95 which assures excellent internal consistency.

#### Foreign Language Test Anxiety Scale

To analyze the efficacy of PB on reducing the TA levels of participant students, FLTAS was implemented to the groups before and after the implementation. The scale aimed at examining the processes foreign language learners go through when dealing with language exams. Overall, FLTAS included 18 items and consisted of a 5-point Likert Scale design measuring the participants’ responses from 1 (Strongly Disagree) to 5 (Strongly Agree). All items were negatively coded. Some of the sample items were as follows: *My body and hands tremble during foreign language tests. Although my foreign language exams are over, I cannot stop feeling worried. When I take language exams, I often get lost in my thoughts and cannot concentrate*. The scoring of items indicates that the higher scores participants gave, the more anxious they feel toward foreign language evaluative conditions. FLTAS was piloted with a similar group from the Faculty of Health Sciences and the Cronbach’s alpha value was estimated 0.93 highlighting high internal consistency.

#### Listening and Reading Comprehension Tests

In order to evaluate the efficacy of PB implementation on participants’ listening and reading comprehension abilities, two tests were administered to study groups before (pre-test), and after (post-test) the implementation. The pre-test is named as Listening and Reading Ability Test while the post test is entitled as Listening and Reading Achievement test. The tests were prepared by the testing office of the institution and were based on the content covered in the textbook. The listening items were based upon dialogs that were authentically constructed and recorded by the testing office of the institution while the reading items were based on passages selected by the same testing unit. The listening tracks were three and a half minutes long and the reading passages were approximately 300 words long. The reading tests consisted of 15 items in total while the listening tests were composed of 10 items. The reading section included matching paragraphs, true or false, and vocabulary in context questions whereas the listening section consisted of filling the missing information and true/false items. Each item in both tests was scored on 1–4. The overall scores for the listening and the reading comprehension tests were 40 and 60, respectively. The reading test items measured the students’ comprehension skills through asking question types that required finding the gist, scanning, and reading for details. The listening test items evaluated the comprehension skills such as scanning and reading for details. The Cronbach’s alpha values for the listening comprehension section in pre- and post-test s were 0.86 and 0.85, respectively, whilst the Cronbach’s alpha value for the reading pre- and post-test was 0.90 showing high consistency.

#### Semi Structured Interviews

Semi-structured interviews (SSIs) were conducted with eight volunteering participants from the experimental group by utilizing a question set of eight items after the PB implementation ended. Some of the semi-structured interview questions were as follows: *Could you describe the general atmosphere in the classroom when pranayama was practiced? How did you feel before, during and after pranayamic breathing exercises? If you had the chance, would you change anything about the breathing exercises*? Each interview roughly took 10–15 min and a couple of probing questions were posed depending on the flow of the inquiries. The interviews were audio recorded and to ensure the validity of the interviews, the transcriptions of what subjects stated were shared with the participants. To establish the inter-rater reliability, the main themes were analyzed by two expert teachers in the field of ELT. The inter-reliability of the discovered themes was found to be 0.84, which indicated a strong level of agreement between two raters.

#### Teacher’s Reflective Journal

In the present study, the teacher’s reflective journal (TRJ) was used to track down the implementation of PB exercises, spot the challenges experienced by the instructor and the participants, and guide the process of application thoroughly. The journal was kept weekly by the instructor in order to enrich the qualitative aspect of the study. TRJ also helped the instructor keep track of individual progress of the participants and tailor the breathing techniques when needed.

### Procedure

#### Data Collection

All of the processes including the implementation of PB and the classes was conducted online as the institution, in which the study was employed, shifted to online education due to COVID-19 pandemic outbreak at the time of the study. Initially, a brief theoretical introduction of the study was provided to the participants. After obtaining the consents, a set of questions to collect demographic information about the participants and their physical and psychological wellbeing was distributed in an online platform. ELLAS, FLTAS and the listening and the reading ability pre-test were given prior to the beginning of the implementations. The groups did not receive any kind of PB implementation prior to the administration of the pre-test and pre-scales. NSP and BP were implemented interchangeably to the experimental group over a 7-week span, on a weekly basis. The implementation started with NSP first week and was switched to BP in the following week. Each week the techniques were interchanged in order to provide a variety of practices and avoid repetitive nature of the techniques. Both techniques were employed 5 min for the first 4 weeks of the study and then were gradually increased to 10 min per each class hour. Depending on the flow of the lessons, the breathing practices were employed when found necessary such as at the beginning, at the end and before and after heavy language tasks to alleviate anxious feelings, energize and cool down the learners. The general flow of the NSP and BP implementations was as follows:

All of the participants in the experimental group were requested to sit comfortably and maintain the correct spine position. The practitioner, who was one of the researchers of the current study and a certified breathing instructor at the same time, asked participants to shut their eyes slowly. They were asked to notice their normal breathing patterns and practiced a few neck, wrist, arm and shoulder stretches. After the stretches and the relaxation, the participating students followed their breathing instructor’s guiding. The practices started off with 5 min per each class hour and were gradually extended to 10 min. Below a sample technique of NSP is illustrated:

1. Close your right nostril gently with your right thumb.
2. Breath in through your left nostril. Observe your breathing.
3. Hold the breath you took through left nostril. With your little finger or ring finger, seal your left nostril, and open the right one. Exhale slowly through the right nostril.
4. Your left finger remains at the same place. Breath in deeply through the right nostril.
5. Hold your breath again and close your right nostril with your right thumb.
6. Inhale slowly through your left nostril. Keep the same cycle going.

At the end of the PB, both groups were given the same scales and the listening and reading post-test. Additionally, semi structured interviews were conducted to shed further light on findings. Besides, the control group received no PB related treatment and continued having mainstream classes. Both PB sessions and mainstream GE classes were conducted through online learning platforms synchronously.

#### Data Analysis

In order to run a data analysis, the quantitative data were submitted the software program IBM SPSS version 28. Normally distributed data were analyzed through independent samples and paired samples *t-*tests while the nonparametric data were examined through Mann-Whitney *U* and Related Samples Wilcoxon Signed Rank Tests. The qualitative data analysis was based on the grounded theory framework and data saturation laid the groundwork. Open, axial, and selective coding were utilized during the analysis. The data were transcribed by the researchers and codification process was conducted through using Dedoose application tool. Next, re-categorization phase took place and umbrella terms were discovered. After thematization, categorization phase was utilized. Relevant statements found were compared and contrasted with each other, and finalized main themes were grouped with their sub-themes. Member-checking was also conducted during and after the interviews as to ensure obtaining credible results.

## Results

### Foreign Language Learning Anxiety Results

An independent samples *t-*test was administrated to compare the overall FLLA mean scores between the control group and the experimental group in pre- and post-conditions. There was not a significant difference in the overall anxiety mean scores between the control group (*M* = 89.0, *SD* = 19.4) and the experimental group (*M* = 86.1, *SD* = 19.8) in pre-condition; *t*(138) = 0.8, *p* = 0.393. However, a statistically significant difference was present between the control group (*M* = 87.7, *SD* = 18.2) and the experimental group (*M* = 82.3, *SD* = 13.5) in post-test condition; *t*(138) = 1.9, *p* = 0.048 (see Table 1).

**TABLE 1:** English Language Learning Anxiety Scale (ELLAS) independent samples *T*-test results.

		*M*	SD	*t*	d*f*	Sig.
Pre	Control	89.0	19.4	0.85	138	0.393
	Experimental	86.1	19.8			
Post	Control	87.7	18.2	1.99	138	0.048*
	Experimental	82.3	13.5			

*p = 0.048, p < 0.05.

Furthermore, a paired sample *t*-test demonstrated that the control group did not have a statistically significant difference in overall FLLA scores in pre- (*M* = 89.0, *SD* = 19.4) and post- (*M* = 87.7, *SD* = 18.2) conditions *t*(69) = 1.3, *p* = 0.172. Although a -1.3 decrease was observed in the overall mean scores, which could be linked to the duration of the study and the mainstream foreign language classes’ cooperative nature, the difference was not statistically significant (*p* = 0.172, *p* > 0.05). The experimental group, however, had a statistically significant difference in the overall English language learning anxiety levels before (*M* = 86.1, *SD* = 19.8) and after (*M* = 82.3, *SD* = 13.5) the PB implementation; *t*(69) = 2.1, *p* = 0.033 (see Table 2).

**TABLE 2:** English Language Learning Anxiety Scale paired sample *T*-test results.

		*M*	SD	*t*	d*f*	Sig
Control	Pre	89.0	19.4	1.38	69	0.172
	Post	87.7	18.2			
Experimental	Pre	86.1	19.8	2.17	69	0.033*
	Post	82.3	13.5			

*p = 0.033, p < 0.05.

### Foreign Language Test Anxiety Results

An independent-samples *t*-test was applied to compare the foreign language TA mean scores of the control and experimental groups before the PB implementation. There was not a statistically significant difference between the control group (*M* = 61.6, *SD* = 13.4) and experimental group (*M* = 58.2, *SD* = 12.1) in pre-condition; *t*(138) = 1.5, *p* = 0.114. More specifically, the control group and experimental group FLLA levels did not differ prior to the implementation (*p* = 0.114, *p* > 0.05). A statistically significant difference, however, was deduced between the control group (*M* = 60.0, *SD* = 12.2) and the experimental group (*M* = 55.4, *SD* = 13.4) TA mean scores after the PB implementation; *t*(138) = 2.0, *p* = 0.038 (see Table 3).

**TABLE 3:** Foreign Language Test Anxiety Scale (FLTAS) independent samples *T*-test results.

		*M*	SD	*t*	d*f*	Sig.
Pre	Control	61.6	13.4	1.58	138	0.114
	Experimental	58.2	12.1			
Post	Control	60.0	12.2	2.09	138	0.038*
	Experimental	55.4	13.4			

*p = 0.038, p < 0.05.

The paired sample *t*-test results for foreign language TA illustrated that there was not a statistically significant difference in the TA mean scores before (*M* = 61.6, *SD* = 13.4) and after (*M* = 60.0, *SD* = 12.2) the implementation *t*(69) = 0.9, *p* = 0.357. Although a −1.6 decrease was seen in the overall mean scores of the control group, which could be attributed to the nature of mainstream classes, this difference was not statistically significant (*p* = 0.357, *p* > 0.05). The experimental group, on the other hand, saw a statistically significant difference in pre- (*M* = 58.2, *SD* = 12.1) and post- (*M* = 55.4, *SD* = 13.4) conditions; *t*(69) = 2.3, *p* = 0.023 (see Table 4).

**TABLE 4:** Foreign Language Test Anxiety Scale (FLTAS) paired sample *T*-test results.

		*M*	SD	*t*	d*f*	Sig
Control	Pre	61.6	13.4	0.92	69	0.357
	Post	60.0	12.2			
Experimental	Pre	58.2	12.1	2.32	69	0.023*
	Post	55.4	13.4			

*p = 0.023, p < 0.05.

### Listening and Reading Comprehension Test Results

Findings for listening and reading comprehension skills revealed that a significant difference did not exist between the listening and reading comprehension test (LRCT) scores of the control and experimental groups before the implementation of PB (*z* = 1.6, *p* = 0.102, *z* = 0.9, *p* = 0.324, *respectively*). The mean differences in LRCT scores between the control and the experimental group was not statistically different (*p* = 0.102 × *listening*, *p* = 0.324, *p* > 0.05 × *reading*) before the implementation. The Mann-Whitney *U* Test findings displayed a statistically significant difference between the control group and the experimental group mean scores in both tests applied after the implementation (*z* = 2.5, *p* = 0.010, *z* = 2.1, *p* = 0.031, *respectively*). The results pointed out that the mean score difference in post-tests between the control and the experimental group was statistically significant (*p* = 0.010 × *listening*, *p* = 0.031, *p* < 0.05 × *reading*). In other words, there was an increase in the listening and reading test scores of the participants after practicing PB (see Table 5).

**TABLE 5:** Listening and Reading Comprehension Tests (LRCT) Mann-Whitney *U* test results.

		Mann-Whitney *U* Test		
		*U*	*Z*	Asymp, Sig.
Pre-Listening	Control	2,063.5	1.632	0.102
	Experimental			
Post-Listening	Control	1,840.5	2.566	0.010*
	Experimental			
Pre-Reading	Control	2,215.5	0.986	0.324
	Experimental			
Post-Reading	Control	1,935.0	2.155	0.031*
	Experimental			

*p = 0.010, p = 0.031, p < 0.05.

Results of LRCTs indicated a non-statistically significant difference in mean scores of control group in pre- and post-conditions (*z* = 0.5, *p* = 0.594, *z* = 1.5, *p* = 0.118, *respectively*). Although gains were observed in the means, differences were not significant (*p* = 0.594 × *listening*, *p* = 0.118 × *reading, p* > 0.05). Put differently, the mainstream GE classes did not have a statistically significant influence on the listening comprehension test scores. However, when Table 6 was analyzed for the experimental group, a statistically significant change was observed between pre- and post-test LRCT scores (*z* = 2.0, *p* = 0.041, *z* = 2.7, *p* = 0.006, *respectively*). Both test mean scores witnessed a statistically significant difference in before and after breathing implementation (*p* = 0.041, *p* = 0.006, *p* < 0.05). Stated differently, using PB in GE classes enabled a significant improvement in the listening and reading comprehension skills of the participants (see Table 6).

**TABLE 6:** Listening and reading comprehension tests Wilcoxon signed ranks test results.

			Pre- post-test statistics^a^
Listening achievement	Control	*Z*	0.533^b^
		Asymp, Sig (2-tailed)	0.594
	Experimental	*Z*	2.038^b^
		Asymp, Sig (2-tailed)	0.041
Reading achievement	Control	*Z*	1.560^b^
		Asymp, Sig (2-tailed)	0.118
	Experimental	*Z*	2.765^b^
		Asymp, Sig (2-tailed)	0.006

*^a^Wilcoxon signed ranks test.*

*^b^Shows significance.*

### Students’ Interview Results

The results gathered from the SSIs showed that after PB, the participants witnessed salient changes in their anxiety level, noticed a better learning atmosphere and had positive changes in their daily lives Ultimately, the overall findings were categorized into three main themes along with a total of six sub-themes (see Table 7).

**TABLE 7:** Semi-structured interview results.

Main themes	Subthemes
(1) Changes observed in anxiety levels	(1a) Lessened fear of foreign language learning (1b) Decrease in test anxiety
(2) Changes observed in general class atmosphere	(2a) Engagement in classes (2b) Peaceful learning environment
(3) Changes observed in daily life	(3a) Regulated sleeping patterns (3b) Improved breathing patterns

#### Changes Observed in Anxiety Levels

This main theme pertaining to the integration of the PB techniques into GE classes stood out as the most salient theme. The participants commented on the changes observed in their FLLA and TA levels and these comments were categorized into two sub-themes.

##### Lessened fear of foreign language learning

The respondents stated seeing positive changes toward their language learning processes. All uttered statements that indicated shrunken worrisome feelings after being introduced to pranayama. Below, one of the excerpts is highlighted in relation to alleviated apprehension:

*…I like pranayama because it is interesting. I think it helps me speak English more. I am feeling more relaxed.*

##### Decrease in test anxiety

All participants commented on how pranayama aided their overall emotional status during testing environments. They particularly emphasized previous apprehensive feelings and uttered rather promising results pertaining to TA. The overall trend was toward the positive efficacy of pranayama since it helped learners concentrate more and feel more alleviated. The following excerpt reflects this observation.

*… I hate English tests. Now, I like English. I do the techniques we are learning, and I can concentrate more.*

#### Changes Observed in General Class Atmosphere

The second most dominant theme was relevant to the in-class alterations remarked by the participants of the study. The majority of the participants stated that learning a foreign language in an environment where tranquility was present helped them relax and have fun. No contradictory themes were discovered. Relating to the aforementioned changes, two sub-themes presented below were deduced.

##### Engagement in classes

Almost all participants stated feeling more engaged and interested in GE classes. They shared some personal experiences regarding the topic in particular and reported feeling more interested in foreign language classes than their other courses as supported in this excerpt:

*… English with pranayama is more fun and interested than other lessons. I was very shy first lesson but now I am more active.*

##### Peaceful learning environment

All participants commented on how peaceful the learning environment was and how relaxed they felt. They were comfortable since their learning environment included peace and empathy as illustrated in this viewpoint:

*… I had so much fun with English classes. Breathing is very useful. Our class had peace and empathy.*

#### Changes Observed in Daily Life

The third main theme was the least dominant one; however, the findings presented in this theme were rather unexpected and different from the initial goals of the study. Four participants mentioned their observations on how their sleeping cycles and sleep routines changed after PB while three participants reported seeing some changes in their daily breathing patterns which are presented in the next section.

##### Regulated sleeping patterns

Four participants experienced noticeable transitions in their sleeping patterns. Some participants suffering from perceived mild sleep disorder especially stressed out witnessing regulated patterns of sleep. Overall, participants were contented with the changes they saw through practicing pranayama in and outside the classes. The following excerpt could confirm the aforementioned findings:

*… I have big sleep problems. I couldn’t sleep easy. I think breathing is helping. I sleep fast and wake up happy.*

##### Improved breathing patterns

Only three participants reported feeling changes in their breathing habits expressing how they felt after practicing pranayama. They felt happier and more energized during the day as presented in this excerpt:

*… I think I breathed wrong before. I used my mouth always. Now I use my nose, I feel more energetic.*

### Teacher’s Reflective Journal Results

After analyzing the TRJ, the findings were classified into five main themes, which were (a) limited class hours, (b) overcrowded online classes, (c) pacing and curriculum restrictions, (d) issues with online teaching, (e) positive attitudes toward implementations.

#### Limited Class Hours

It was found out that the limited class hours caused several drawbacks with the applications of PB. The learners had difficulties in remembering the previous week’s PB technique. The following excerpts reflect this finding:

*… It is sometimes tough for me to get the learners ready for the breathing exercises. Some forget the techniques and the steps. We need more class hours that are distributed equally to the days of the week.*

#### Overcrowded Online Classes

Due to COVID-19 outbreak and the decision to shift education into online platforms, online classes were rather packed with learners. The reflections of the instructor revealed that practicing mindfulness a high number of students could be possible yet demanding. Considering this finding, the instructor said:

*…I have a high population problem which might be difficult to control.*

#### Pacing and Curriculum Restrictions

The restrictions faced concerning the flexibility of the syllabus caused further challenges. Although the institution was not extremely strict with pacing, the instructors had to cover the intended main coursebook pages. The above-noted troubles could be demonstrated in the following excerpt:

*… Being required to cover fixed pages of the mainstream book and following the pacing is overwhelming with the population of the class and the class hours.*

#### Issues With Online Teaching

The reflections of the breathing instructor displayed further problems occurring due to the nature of the online learning environments and the online teaching policies of the institution. The biggest concern was the fact that the learners did not have to turn on their microphones and cameras unless they wished to do so. In addition, the online teaching tools sometimes created connection problems and delays as presented in the following statements:

*… There are some issues occurring such as learners’ having technical problems and delays. Some learners are shy and do not want to turn on their cameras during practices which might be troublesome.*

#### Positive Attitudes Toward Implementations

The last theme was related to constructive approaches that the learners had toward PB. This stance eased the implementation procedure, and the participants were able to see the benefits. The following except supports this finding:

*… It is nice to see that learners enjoy PB. They are motivated and are getting more collaborative in the classes.*

The first research question targeted at measuring the impact of PB methods on the FLLA of the undergraduate GE learners. The previously mentioned results indicated that a statistically significant difference was present between the FLLA mean scores of the experimental and control group participants after practicing pranayama (*p* < 0.05). Put differently, the integration of pranayama into GE classes had a positive effect on lowering the students’ FLLA. The second research question aimed at scrutinizing the effect of PB applications on the TA of the undergraduate foreign language learners. The quantitative results demonstrated that there was a statistically significant difference between the experimental group and the control group after the implementations (*p* < 0.05). The target of the third research question was to analyze the impact of PB applications on the foreign language listening and reading comprehension skills. Analyses showed that the gain scores in LRCTs were only statistically significant in the experimental group after the PB implementation. In other words, it was discovered that implementing pranayama in GE classes improved the listening and reading comprehension skills of the foreign language learners to a considerable extent (*p* < 0.05). The last research question explored the insights that could be gained into the integration of PB in GE learning settings with respect to the participants’ and the breathing instructor’s perspectives. Semi-structured interview findings demonstrated that foreign language learners were in favor of PB practices and considered it a useful medium whereas the majority of the data coming from the teacher’s journal showed that there were several areas to consider first before implementing pranayama in foreign language classroom settings.

## Discussion

The aim of this study was to investigate the impact of pranayama breathing as positive psychology exercise on FLLA and TA levels. The study also attempted to find out the changes in the foreign language listening and reading comprehension skills of undergraduate students after pranayama breathing as well as explore the participants’ and the breathing instructor’s insights regarding the integration of pranayama into GE classes. The overall results gathered through FLTAS, ELLAS, listening and reading achievement tests, SSIs and TRJ showed that learners’ foreign language apprehension and TA were reduced, listening and reading comprehension skills were enhanced, and the implementation of pranayama was favored as a useful medium.

The differences in the levels of FLLA and TA after pranayama implementation could be attributed to the peaceful nature of the learning environments where empathy, optimism and partnership nourished. Similar findings were observed in the studies of Calp (2020) and Monshat et al. (2013) emphasizing that tranquility and positive thinking enabled individuals feel valued, optimistic and at ease. In the case of language learning process, yoga—in its deepest essence—is particularly useful since it orientates the learners to focus on the present moment and direct their attention at their endeavors (Morgan, 2011). By the same token, higher levels of self-awareness and regulation are keenly connected to enriched capabilities in the social-emotional functions (Sahdra et al., 2011). In consequence, enhanced social-emotional capabilities are associated with a better mental welfare (Diekstra et al., 2008). Therefore, it could be plausible in the current study that PB allowed learners to feel safe in learning environments and center their attention on the language acquisition process rather than being stuck with unpleasant feelings. Previously, it was proven that learners with higher levels of mindfulness had lower levels of TA (Cunha and Paiva, 2012). It was also found in a different study that pranayama improved brain activities, attention and awareness as well as lessening anxious feelings (Novaes et al., 2020). The current study also corresponds to Nemati’s (2013) study in which the researcher found that pranayama mitigated TA levels of foreign language learners even though the context, the research tools, and the implementation phases differed significantly from ours.

Enhanced listening and reading comprehension scores after practicing breathing exercises might be linked to pranayama equipping learners with higher executive functioning skills. Both listening comprehension and reading comprehension are interactive procedures (Meniado, 2016) influenced by a variety of factors such as lexical command, memory capacity, concentration, familiarity with the context, language expertise, motivation, and usage of metacognitive skills and strategies (Yildiz and Albay, 2015). In a study by Powell et al. (2008), it was discovered that yoga had a significant impact on regulating self-confidence levels, usage of self-talk and increased communication skills. It was also found out that breathing could be beneficial in enhancing attention levels and soothing the worrisome mind (Jensen et al., 2012). Moreover, Mrazek et al. (2013) highlighted in their study that mindfulness-based activities decreased the mind wandering behavior of the learners due to higher levels of concentration during tasks. Therefore, it is plausible that pranayama impacted learners’ foreign language listening and reading comprehension abilities positively by affording them more concentration chances. PB was able to increase their listening and reading comprehension scores in this study; however, the effect of pranayama on foreign language learners’ listening and reading comprehension skills should be investigated further, as studies probing this claim are almost non-existing.

An additional factor carrying an important role in enhancing learners’ listening and reading comprehension skills could be related to the reduced levels of overall FLA, TA and increased motivation levels. This claim resonates with Nemati’s (2013) showing a negative correlation between test apprehension and foreign language exam performance. In addition, Nidich et al. (2011) explored the effect of mindfulness-based practices on improving academic achievement of the learners concluding that learners practicing meditation for 3 months scored better than the control group in English and Mathematic performance tests. It is also known that mindfulness-based practices could influence comfort and enjoyment levels during task completion processes (Kee and Liu, 2011). Considering that anxiety has a negative impact on foreign language listening and reading comprehension skills (Bloomfield et al., 2010), it would not be unfair to presume that PB enhanced the listening and reading comprehension scores of the learners through alleviating FLA and TA levels.

The qualitative results demonstrated that participants witnessed changes observed in anxiety levels, in general class atmosphere and in daily life. These changes might be credited to ameliorated living standards and an overall decline in stress levels. Although based on contemplative practices that are not pranayama centered, Scida and Jones’s (2017) bears a direct resemblance to the qualitative findings of the study as the researchers too found that language learners regarded the practices as quite useful and felt calmer and relaxed during language classes. It was also previously proved that yoga and pranayama enhance the quality of life and sleeping in general (Önder, 2019). In a similar vein, a former study conducted by Wall (2005) regarding the usefulness of mindfulness-based practices in educational settings confirmed that improved sleeping patterns and a general relaxed state could be observed by both learners and their instructors when mindfulness exercises were performed. Additionally, Hjeltnes et al. (2015) discovered that application of mindfulness-based practices enabled learners to stay focused while learning, leave the fear away, and accept the self when dealing with struggles. The abovementioned results resonate with this study as the current study participants reported feeling more relaxed, using breathing techniques before the examinations and finding pleasure in the learning process after being introduced to pranayama.

Although there were some challenges recorded by the breathing instructor regarding the implementation of pranayama in foreign language classes, the language learners were quite positive toward the practices and did not focus on the problems that stemmed from the high population of the class, the technical problems, pacing and the limited class hours. The overall positive tendency of the learners toward the implementations could be attributed to the fact that mindfulness-based practices allowed learners to be optimistic and have well-constructed prosocial behaviors (Schonert-Reichl and Lawlor, 2010). Therefore, the integration of PB helped learners during the foreign language learning process; however, the implementation of PB needs to be well planned and contemplated on carefully.

Ultimately, the results indicated that by centering the attention on the act learning and becoming aware of the present moment, language learners could control the apprehensive feelings more and experience a sophisticated way of learning. The current study results are highly relevant to Krashen’s (1982) theory which emphasized the importance of lowering the affective variables in foreign language learning environments so that learners could feel safe in their learning atmospheres. The obtained results are also aligned with the valued concepts found in positive psychology discipline such as the concept of flow, which is being entirely engaged and present at the current moment and maintaining control over one’s self (Ivtzan and Papantoniou, 2014). Constituting another focal interest in positive psychology, overall well-being and optimal self-actualization functions nourished when integrating pranayama into foreign language learning settings (Ahmadi et al., 2016).

Last but not least, it is worth noting that PB exercises should not simply be considered as a shortcut to quickly amending the very existence of FLA and TA or to improving foreign language learners’ listening and reading comprehension skills. The nature of the pranayama is indeed linked to the control of one’s mind and body and therefore must not be treated as a discrete discipline when one pursues the goal to exploit its potential benefits. For those wishing to track the efficaciousness of such a discipline, there is a need to acknowledge and respect the general essence of yoga practice, the need to recognize one’s own capacities and boundaries, and the need to realize how persevering one can be. Concisely, the integration of pranayama into foreign language learning settings should be approached in a very delicate manner and with a clear mindset if the intent is unraveling the discipline’s true potentials.

## Conclusion

The present study significantly contributes to the literature through scrutinizing the impact of the positive psychology technique of pranayama on alleviating the foreign language learning and TA levels of learners, as well as touching upon the changes in the foreign language listening and reading comprehension levels of undergraduate language learners. The study results revealed that the undergraduate English language learners’ FLLA and TA were reduced, and learners improved their foreign language listening and reading comprehension skills after PB implementation. Besides, it was evinced that foreign language learners were in favor of the breathing practices as they were able to see explicit changes in their anxiety levels and in their approach to language learning process. Interestingly enough, a few learners encountered changes in their sleeping habits and daily breathing patterns. Last not but least, even though the integration of pranayama into foreign language learning environments was found to be challenging by the breathing instructor, a high majority of foreign language learners thrived in their foreign language learning process and found the breathing techniques quite useful and enjoyable.

Although the current study accomplished its goals with the help of a variety of data collection tools and rigorous data analysis process, some limitations regarding the application of the study need to be highlighted. To begin with, the size of the sample (*N* = 140) was not sufficient enough for a study that was quite novice in the field of ELT. Had the study involved more participants both for the control group and the experimental group, the study could have ensured further well-grounded and well-founded results which could have also helped with the generalizability of the sample. A second limitation was the duration of the study lasting for 7 weeks. Even though the effectiveness of PB in alleviating anxiety was previously highlighted in other contexts, the attempt to analyze the effects on FLLA, TA and the advancements in foreign language listening and reading comprehension was rather new; therefore, carrying out a more longitudinal study might have resulted in a more elaborative understanding of the matter. Moreover, only students from the Faculty of Health Sciences were found to be convenient for the study. Analyzing learners from various departments and faculties in the institution or from other universities might have promoted a more comprehensive external validity. The fourth limitation of the study was the foreign language skills assessed, which were the listening and the reading comprehension skills. The study only focused on the changes in the foreign language listening and reading skills due to the administrative issues and COVID-19 pandemic. Had the study analyzed other language skills such as foreign language speaking and writing, the findings could have been more comprehensive. Ultimately, some participants were already familiar with basic and technical breathing exercises, which might be linked to the fact that participants were selected from the Faculty of Health Sciences. Since such variables could have the potential to affect the course of the studies, further studies must dwell upon obtaining demographic and overall health status information.

Concisely, incorporating pranayama necessitates a large amount of planning and preparation time. It is recommended that the foreign language progress of learners needs to be tracked and their needs have to be met, with smaller number of students first, and then, with a larger group. Since there are almost no studies conducted in ELT, pranayama should be practiced with caution, getting enough expertise in practicing the breathing exercises. A high majority of learners may not be familiar with pranayama or not be ready to practice mindfulness techniques; thus, motivating learners and grounding them in the philosophy of yoga are essential. Further studies including a myriad of different contexts, participants and instruments are desperately called for to truly unravel the uncharted effects of pranayama on facilitating foreign language learning process through mitigating negative feelings and emotions.

Concisely, the results divulged that undergraduate English language learners’ FLA and TA were mitigated after practicing pranayama. Learners considered PB as a practical medium in ameliorating the anxious feelings, fostering the classroom learning environments, and regulating the daily habits. Therefore, integrating pranayama into GE classes could be regarded as an empowering attempt to provide learners with safer learning environments.

## Data Availability Statement

The original contributions presented in the study are included in the article/supplementary material, further inquiries can be directed to the corresponding author.

## Ethics Statement

The studies involving human participants were reviewed and approved by Bahcesehir University Ethics Committee. The patients/participants provided their written informed consent to participate in this study.

## Author Contributions

MT conceived the ideas, collected, analyzed the data, and will act as the guarantor. EM drafted the first manuscript, contributed to the study design, and the interpretation of results with KS. All authors contributed to the article and approved the submitted version.

## Conflict of Interest

The authors declare that the research was conducted in the absence of any commercial or financial relationships that could be construed as a potential conflict of interest.

## Publisher’s Note

All claims expressed in this article are solely those of the authors and do not necessarily represent those of their affiliated organizations, or those of the publisher, the editors and the reviewers. Any product that may be evaluated in this article, or claim that may be made by its manufacturer, is not guaranteed or endorsed by the publisher.

## References

[B1] Ahmadi S. Ahmadi S. Kheirandish A. (2016). The effectiveness of yoga practice on positive psychology constructs: meaning in life, gratitude and marital intimacy. *Int. J. Psychol.* 10 105–126.

[B2] Anand A. Patwardhan K. Singh R. N. Awasthi H. H. (2018). Effects of pranayama on mental health and physical fitness in healthy university students. *Yoga Mimamsa.* 50 27–30. 10.4103/ym.ym_15_17

[B3] Aydın S. Yavuz F. Yeşilyurt S. (2006). Test anxiety in foreign language learning. *J. Soc. Sci. Inst.* 9 145–160.

[B4] Bloomfield A. Wayland S. C. Rhoades E. Blodgett A. Linck J. Ross S. (2010). *What Makes Listening Difficult? Factors Affecting Second Language Listening Comprehension.* College Park, MD: University of Maryland Center for Advanced Study of Language.

[B5] Brown K. W. Ryan R. M. Creswell J. D. (2007). Mindfulness: theoretical foundations and evidence for its salutary effects. *Psychol. Inq.* 18 211–237. 10.1080/10478400701598298

[B6] Brown R. P. Gerbarg P. L. (2005). Sudarshan kriya yogic breathing in the treatment of stress, anxiety, and depression: part II—clinical applications and guidelines. *J. Altern. Complement. Med.* 11 711–717. 10.1089/acm.2005.11.711 16131297

[B8] Butzer B. Bury D. Telles S. Khalsa S. B. S. (2016b). Implementing yoga within the school curriculum: a scientific rationale for improving social-emotional learning and positive student outcomes. *J. Child Serv.* 11 3–24. 10.1108/JCS-10-2014-0044

[B7] Butzer B. Ahmed K. Khalsa S. B. S. (2016a). Yoga enhances positive psychological states in young adult musicians. *Appl. Psychophysiol. Biofeedback* 41 191–202. 10.1007/s10484-015-9321-x 26721471 PMC4860116

[B9] Calp Ş. (2020). Peaceful and happy schools: how to build positive learning environments? *Int. Electron. J. Elementary Educ.* 12 311–320. 10.26822/iejee.2020459460

[B10] Craske M. G. Stein M. B. (2016). Anxiety. *Lancet* 388 3048–3059. 27349358 10.1016/S0140-6736(16)30381-6

[B11] Cunha M. Paiva M. J. (2012). Text anxiety in adolescents: the role of self-criticism and acceptance and mindfulness skills. *Span J. Psychol.* 15 533–543. 10.5209/rev_SJOP.2012.v15.n2.38864 22774427

[B12] Dhanvijay A. D. Chandan L. (2018). Effect of nadi shuddhi pranayama on perceived stress and cardiovascular autonomic functions in 1st year undergraduate medical students. *Natl. J. Physiol. Pharm. Pharmacol.* 8 898–902. 10.5455/njppp.2018.8.0205515022018

[B13] Diekstra R. Sklad M. Gravesteijn C. Ben J. Ritter M. D. (2008). “Teaching social and emotional skills world-wide. a meta-analytic review of effectiveness,” in *Social and Emotional Education: An International Analysis*, eds Pablo F. B. Clouder C. (Santander: Fundacion Marcelino Botin), 255–312.

[B14] Dörnyei Z. (2005). *The Psychology of the Language Learner: Individual Differences in Second Language Acquisition.* Mahwah, NJ: Lawrence Erlbaum Associates Publishers.

[B15] Elkhafaifi H. (2005). Listening comprehension and anxiety in the Arabic language classroom. *Mod. Lang J.* 89 206–220. 10.1111/j.1540-4781.2005.00275.x

[B16] Gupta A. Sinha S. Pribesh S. Maira S. (2014). A fresh breath into student achievement: pranayama and educational outcomes. *Int. J. Hum. Soc. Sci. Invent.* 3 38–46.

[B17] Halat S. Özbay M. (2018). The examination of listening anxiety level of the students who learn turkish as a foreign language. *Univers J. Educ. Res.* 6 1–10. 10.13189/ujer.2018.060101

[B18] Hashemi M. (2011). Language stress and anxiety among the English language learners. *Proc. Soc. Behav. Sci.* 30 1811–1816. 10.1016/j.sbspro.2011.10.349

[B19] Hepburn S. J. McMahon M. (2017). Pranayama meditation (yoga breathing) for stress relief: is it beneficial for teachers? *Aust. J. Teach. Educ.* 42 142–159. 10.14221/ajte.2017v42n9.9

[B20] Hjeltnes A. Binder P. E. Moltu C. Dundas I. (2015). Facing the fear of failure: an explorative qualitative study of client experiences in a mindfulness-based stress reduction program for university students with academic evaluation anxiety. *Int. J. Qual. Stud. Health Well Being* 10:27990. 10.3402/qhw.v10.27990 26297629 PMC4545197

[B21] Horwitz E. (2001). Language anxiety and achievement. *Annu. Rev. Appl. Linguist.* 21 112–127. 10.1017/S0267190501000071

[B22] Horwitz E. K. Young D. (1991). *Language Anxiety: From Theory and Research to Classroom Implications.* Englewood Cliffs, NJ: Prentice Hall.

[B23] Horwitz E. K. Horwitz M. B. Cope J. (1986). Foreign language classroom anxiety. *Mod. Lang J.* 70 125–132. 10.2307/327317

[B24] Ivtzan I. Papantoniou A. (2014). Yoga meets positive psychology: examining the integration of hedonic (gratitude) and eudaimonic (meaning) wellbeing in relation to the extent of yoga practice. *J. Bodyw. Mov. Ther.* 18 183–189. 10.1016/j.jbmt.2013.11.005 24725784

[B25] James A. (2009). *Effect of Select Yogasanas, Pranayama and Meditation on Biochemical, Physiological, and Psychological Variables of Male Students Ph. D, Thesis.* Department of Physical Education, Pondicherry University.

[B26] Jensen P. Stevens P. Kenny D. (2012). Respiratory patterns in students enrolled in schools for disruptive behaviour before, during, and after yoga nidra relaxation. *J. Child Fam. Stud.* 21 667–681.

[B27] Joshi A. Singh M. Joshi S. Singla B. B. (2011). Enhanced wellbeing amongst engineering students through nadi shodhana pranayama (alternate nostril breathing) training: an analysis. *Sch. Dr. Stud.* 3 112–120.

[B28] Kee Y. H. Liu Y. (2011). Effects of dispositional mindfulness on the self-controlled learning of a novel motor task. *Learn. Individ. Differ.* 21 468–472. 10.1016/j.lindif.2011.01.009

[B29] Kharya C. Gupta V. Deepak K. K. Sagar R. Upadhyav A. Kochupillai V. (2014). Effect of controlled breathing exercises on the psychological status and the cardiac autonomic tone: sudarshan Kriya and Prana-Yoga. *Indian J. Physiol. Pharmacol.* 58 210–220. 25906603

[B30] Krashen S. D. (1982). *Principles and Practice in Second Language Acquisition.* Oxford: Pergamon.

[B31] Kumar N. Pradhan B. (2017). Immediate role of two yoga-based breathing technique on state anxiety in patients suffering from anxiety disorder: a self as control pilot study. *Int. J. Yoga Philosop Psychol. Parapsychol.* 5 18–23. 10.4103/ijny.ijoyppp_9_16

[B32] Kuppusamy M. Dilara K. Ravishankar P. Julius A. (2017). Effect of bhramari praṇayama practice on pulmonary function in healthy adolescents: a randomized control study. *Anc. Sci. Life.* 36 196–199. 10.4103/asl.ASL_220_16 29269971 PMC5726186

[B33] Kuppusamy M. Kamaldeen D. Pitani R. Amaldas J. Shanmugam P. (2018). Effects of bhramari pranayama on health–a systematic review. *J. Tradit. Complement. Med.* 8 11–16. 10.1016/j.jtcme.2017.02.003 29321984 PMC5755957

[B34] Levine M. (2000). *The Positive Psychology of Buddhism and Yoga: Paths to A Mature Happiness.* Mahwah, NJ: Lawrence Erlbaum.

[B35] Malhotra V. Jha J. P. Jha O. P. Garg R. Itagappa M. Tripathi Y. (2016). Effect of brahmari pranayama on visual reaction time. *J. Evol. Res. Hum. Physiol.* 2 1–3. 10.25078/jyk.v1i1.1537

[B36] Meniado J. C. (2016). Metacognitive reading strategies, motivation, and reading comprehension performance of saudi EFL students. *Engl. Lang Teach.* 9 117–129. 10.5539/elt.v9n3p117

[B37] Middeldorp C. M. Cath D. C. Van Dyck R. Boomsma D. I. (2005). The co-morbidity of anxiety and depression in the perspective of genetic epidemiology. a review of twin and family studies. *Psychol. Med.* 35 611–624. 10.1017/S003329170400412X 15918338

[B38] Monshat K. Khong B. Hassed C. Vella-Brodrick D. Norrish J. Burns J. (2013). A conscious control over life and my emotions: mindfulness practice and healthy young people. a qualitative study. *J. Adolesc. Health.* 52 572–577. 10.1016/j.jadohealth.2012.09.008 23298987

[B39] Morgan L. (2011). Harmonious learning: yoga in the english language classroom. *Eng. Teach. Forum.* 49 2–13.

[B40] Mrazek M. D. Franklin M. S. Phillips D. T. Baird B. Schooler J. W. (2013). Mindfulness training improves working memory capacity and GRE performance while reducing mind wandering. *Psychol. Sci.* 24 776–781. 10.1177/0956797612459659 23538911

[B41] Nemati A. (2013). The effect of pranayama on test anxiety and test performance. *Int. J. Yoga* 6 55–60. 10.4103/0973-6131.105947 23439436 PMC3573544

[B42] Nidich S. Mjasiri S. Nidich R. Rainforth M. Grant J. Valosek L. (2011). Academic achievement and transcendental meditation: a study with at-risk urban middle school students. *Education* 131 556–564.

[B43] Novaes M. M. Palhano-Fontes F. Onias H. Andrade K. C. Lobão-Soares B. Arruda-Sanchez T. (2020). Effects of yoga respiratory practice (Bhastrika pranayama) on anxiety, affect, and brain functional connectivity and activity: a randomized controlled trial. *Front. Psychiatry* 11:467. 10.3389/fpsyt.2020.00467 32528330 PMC7253694

[B44] Önder ÖÖ (2019). Efficacy of yoga and pranayama on sleep disorders. *Sleep Vigil.* 3 1–6. 10.1007/s41782-019-00072-6

[B45] Powell L. Gilchrist M. Stapley J. (2008). A journey of self-discovery: an intervention involving massage, yoga and relaxation for children with emotional and behavioural difficulties attending primary schools. *Emot. Behav. Diffic.* 13 193–199. 10.1080/13632750802253186

[B46] Pramanik T. Sharma H. O. Mishra S. Mishra A. Prajapati R. Singh S. (2009). Immediate effect of slow pace Bhastrika pranayama on blood pressure and heart rate. *J. Altern. Complement Med.* 15 293–295. 10.1089/acm.2008.0440 19249921

[B47] Sahdra B. K. MacLean K. A. Ferrer E. Shaver P. R. Rosenberg E. L. Jacobs T. L. (2011). Enhanced response inhibition during intensive meditation training predicts improvements in self-reported adaptive socioemotional functioning. *Emotion* 11 299–312. 10.1037/a0022764 21500899

[B48] Schonert-Reichl K. A. Lawlor M. S. (2010). The effects of a mindfulness-based education program on pre- and early adolescents’ well-being and social and emotional competence. *Mindfulness* 1 137–151. 10.1007/s12671-010-0011-8

[B49] Scida E. E. Jones J. N. (2017). The impact of contemplative practices on foreign language anxiety and learning. *Stud. Second. Lang Learn. Teach.* 7 573–599. 10.14746/ssllt.2017.7.4.2

[B50] Scovel T. (1978). The effect of affect on foreign language learning: a review of the anxiety research. *Lang Learn.* 28 129–142. 10.1111/j.1467-1770.1978.tb00309.x

[B51] Sellers V. D. (2000). Anxiety and reading comprehension in Spanish as a foreign language. *Foreign Lang Ann.* 33 512–520. 10.1111/j.1944-9720.2000.tb01995.x

[B52] Sengupta P. (2012). Health impacts of yoga and pranayama: a state-of-the-art review. *Int. J. Prev. Med.* 3 444–458. 22891145 PMC3415184

[B53] Shah M. R. Kothari P. H. (2019). Effects of nadi-shodhana pranayama on depression, anxiety, stress and peak expiratory flow rate in post CABG patients: experimental study. *Int. J. Health Sci. Res.* 9 40–45. 10.46858/vimsjpt.2108

[B54] Singleton M. (2010). *Yoga Body: the Origins of Modern Posture Practice.* New York: Oxford University Press.

[B55] Srivastava S. Goyal P. Tiwari S. K. Patel A. K. (2017). Interventional effect of bhramari pranayama on mental health among college students. *Int. J. Indian Psychol.* 4 29–33.

[B56] Suleimenova Z. (2013). Speaking anxiety in a foreign language classroom in Kazakhstan. *Proc. Soc. Behav. Sci.* 93 1860–1868. 10.1016/j.sbspro.2013.10.131

[B57] Tiller J. W. G. (2012). Depression and anxiety. *Med. J. Aust.* 1 28–31. 10.5694/mjao12.10628

[B58] Vhavle S. Rao M. R. Manjunath N. K. Ram A. R. (2017). Effects of a yoga program on health, behaviour and learning ability in school children: a single arm observational study. *Int. J. Altern. Complement. Med.* 5:00138. 10.15406/ijcam.2017.05.00138

[B59] von Wörde R. (2013). Students’ perspectives on foreign language anxiety. *Inquiry* 8 21–40.

[B60] Wall R. B. (2005). Tai chi and mindfulness-based stress reduction in a boston public middle school. *J. Pediatr. Health Care* 19 230–237. 10.1016/j.pedhc.2005.02.006 16010262

[B61] World Health Organization (2017). *Depression and Other Common Mental Disorders: Global Health Estimates (No.WHO/MSD/MER/2017.2).* Available online at: https://apps.who.int/iris/handle/10665/254610 (accessed June 21, 2021).

[B62] Yildiz N. Albay M. (2015). Factors affecting listening comprehension and strategies for listening class. *Int. J. Soc. Sci. Educ. Stud.* 2 20–24.

[B63] Young D. J. (1990). An investigation of students’ perspectives on anxiety and speaking. *Foreign Lang Ann.* 23 539–553. 10.1111/j.1944-9720.1990.tb00424.x

[B64] Yue X. (1996). Test anxiety and self-efficacy: levels and relationship among secondary school students in Hong Kong. *Psychol. Int. J. Psychol. Orient.* 39 193–202.

